# Stress-driven temporal production of phage tail-like particles (tailocins) in *Dickeya dadantii* strain 3937

**DOI:** 10.1038/s41598-025-13158-1

**Published:** 2025-07-26

**Authors:** Marta Sobolewska, Dorota M. Krzyżanowska, Marcin Borowicz, Robert Czajkowski

**Affiliations:** 1 Laboratory of Biologically Active Compounds, Intercollegiate Faculty of Biotechnology, University of Gdańsk and Medical University of Gdańsk, University of Gdańsk, A. Abrahama 58, Gdańsk, 80-307 Poland; 2 Laboratory of Plant Microbiology, Intercollegiate Faculty of Biotechnology, University of Gdańsk and Medical University of Gdańsk, University of Gdańsk, A. Abrahama 58, Gdańsk, 80-307 Poland

**Keywords:** Real-time qPCR, Phage tail-like particles, *Erwinia chrysanthemi*, Bacteriophage, Microbial ecology, Bacteriophages, Environmental microbiology, Virology, Bacterial genes

## Abstract

Tailocins are bacteriocins resembling bacteriophage tails. Previously, we reported the production of tailocins in the plant pathogen *Dickeya dadantii* 3937, the synthesis of which was upregulated upon treatment with mitomycin C. In this study, we investigated how mitomycin treatment over time influences the expression of tailocin-related genes, the accumulation of tailocin particles, and the survival of producer cells. The expression of tailocin P2D1 structural genes peaks two hours after the addition of mitomycin C as measured with an RT-qPCR assay. Simultaneous measurements of tailocin titer revealed that the concentration of the particles in the culture supernatant peaked 6 h after induction and remained stable for at least 18 h. Progressive accumulation of P2D1 that occurred from 2 to 6 h after mitomycin C treatment was associated with a substantial decrease in viable cells of the tailocin-producing strain (ca. 100,000-fold). Decreased cell viability upon tailocin production indicates that they are released from the cells upon cell lysis. Likewise, we found new potent inducers, viz., hydrogen peroxide and antibiotics affecting DNA replication and repair (viz. norfloxacin and ciprofloxacin), that can increase tailocin yield in *D. dadantii* 3937.

**Supplementary Information:**

The online version contains supplementary material available at 10.1038/s41598-025-13158-1.

## Introduction

Approximately 70–80% of sequenced bacterial genomes contain genes of viral origin, suggesting that these viral genes contribute to important phenotypes in the bacterial hosts^1,2^. Some of these genetic elements can encode phage tail-like particles known as tailocins^3^. Tailocins are nanomolecular structures resembling syringes that share evolutionary and morphological features with bacteriophage tails, type VI secretion systems, and extracellular contractile injection systems^4^. These phage tail-like particles are used in both defense mechanisms as well as offensive weapons by bacteria^5^. They are released from bacteria in response to environmental stresses or competitive pressure, acting as precision weapons, targeting and killing closely related bacterial competitors^6^. By eliminating those closely related strains, the producers improve their chances of survival and resource acquisition in various ecological niches^7,8^.

Tailocin production has been described both in Gram-negative and Gram-positive bacteria^9–12^. The producing strains encompass a range of human, animal, and plant pathogens as well as saprophytic bacteria found in diverse environments, including the rhizosphere, bulk soil, within plants, aquatic habitats, as well as in insects, animals, and humans (reviewed in:^13^. We recently described a novel tailocin dickeyocin P2D1 that is produced by the plant pathogenic bacterium *Dickeya dadantii* strain 3937. P2D1 tailocins resemble the tail structures of bacteriophage *Peduovirus* P2. They exhibit bactericidal activity specifically against phylogenetically related bacterial strains without adversely affecting eukaryotic cells in a *Caenorhabditis elegans* killing model^14^.

The genetic cluster encoding the P2D1 tailocin seems to be widely distributed in the genomes of *Dickeya* spp. In our recent screen of 74 complete and high-quality *Dickeya* spp. genomes deposited in the NCBI database, 52 (70% of the genomes) contained tailocin clusters having high homology to the P2D1 cluster (> 70% threshold for coverage and identity)^15^. The widespread presence of P2D1 tailocins in this bacterial group suggests that they provide a competitive advantage during interactions with closely related strains.

Production of tailocins is known to be triggered by the SOS response associated with DNA damage^16^. Still, the exact mechanism of induction and production of phage tail-like particles remains poorly understood for most analyzed strains. Under laboratory settings, the most commonly used inducers of phage tail-like particles are UV radiation and mitomycin C. UV radiation primarily induces reversible thymine dimers in DNA. This activation triggers the cleavage of the LexA repressor, leading to the derepression of SOS-regulated genes, including those involved in the production of tailocins^17^. In contrast, mitomycin C is a DNA crosslinking agent that induces severe and irreversible DNA damage, activating the SOS response^18^. Mitomycin C can cause a stronger and more prolonged SOS response than UV radiation and, therefore, probably stimulates more production of tailocins than UV radiation in vitro^19^.

Among the current knowledge gaps in tailocin biology are details of the environmental conditions under which the production of tailocins is triggered and how these conditions may contribute to the overall success of a particular strain in a given niche^6^. It is plausible that many factors contributing to cellular stress in natural environment may induce tailocin production. However, no comprehensive studies have yet been conducted to explore the in situ induction of tailocins. Additionally, there is limited data on the temporal relationships between the induction of tailocin production and the accumulation of active particles. Such insights are needed to better understand how tailocins can mediate the outcomes of interactions between bacterial strains in natural settings. Therefore, this study aimed to determine the conditions that trigger P2D1 tailocin production in *D. dadantii* strain 3937. Specifically, we explored the mitomycin C concentration-dependent as well as the subsequent time-dependent production of P2D1 tailocin. In turn, the accumulation of P2D1 tailocin was linked to the expression of P2D1 genes upon induction in real time using qRT-PCR. We also assessed other triggers of P2D1 induction and discussed how these inducers could modulate the concentration of P2D1 tailocins under natural environmental conditions.

## Materials and methods

### Bacterial strains, chemical compounds, and growth media

*Dickeya dadantii* strain 3937^20^ and *Musicola paradisiaca* strain IFB 0117 (NCPPB 2511)^21,22^ were grown at 28 °C on either trypticase soya agar (TSA; Oxoid), in trypticase soy broth (TSB; Oxoid), in potato dextrose broth (PDB; Biocorp) or in M9 minimal medium (MP Biomedicals) supplemented with glucose (Sigma-Aldrich) to a final concentration of 0.4%. Liquid cultures were agitated during incubation (120 rpm). To solidify the media, 15 g L^− 1^ bacteriological agar (Oxoid) was added. Soft top agar (STA) was prepared using 30 g of trypticase soy broth (Oxoid) and 7 g of bacteriological agar (Oxoid) per liter. When required, the growth media were supplemented with various concentrations of mitomycin C (Abcam), chloramphenicol (A&A Biotechnology), ampicillin (A&A Biotechnology), ciprofloxacin (Sigma-Aldrich), norfloxacin (Abcam) or hydrogen peroxide (Laboratorium Galenowe Olsztyn, Poland) as described below.

### Induction, purification, and Estimation of Tailocin titer after induction

P2D1 tailocins were induced, purified, concentrated, and quantified as described in^14^. Briefly, *Dickeya dadantii* strain 3937 was grown overnight (ca. 16 h) in TSB at 28 °C with shaking (120 rpm). The cultures were then rejuvenated by diluting them 1:40 in 10 or 100 mL of fresh TSB medium. The diluted culture grew for 2.5 h under the same conditions, reaching approximately 7.5 log colony-forming units (CFU) per milliliter, with a turbidity of 0.8 to 1.0 on the McFarland scale. Such prepared *D. dadantii* cultures were then supplemented with mitomycin C (Abcam, Poland) to a final concentration of 1 µg mL^− 1^ to induce the production of P2D1 tailocins. Following mitomycin C treatment, the cultures were incubated for another 24 h at 28 °C with shaking (120 rpm). 2.2 mL of each post-induction bacterial culture was cleared from bacterial cells through centrifugation (10 min., 8000 RCF, 22 °C). The resulting supernatant was filtered (0.2 μm, PES membrane, Googlab), and 2 mL of the filtrate was transferred to tubes containing polyethylene glycol (PEG-8000, Promega) to achieve the final PEG concentration of 10%. The samples were incubated for 16–20 h at 4 °C with gentle horizontal shaking to precipitate tailocins. The precipitant was harvested by centrifugation (1 h, 16000 RCF, 4 °C). After removing the supernatant, the samples were dried under laminar flow until the remaining liquid evaporated. The resulting tailocins were resuspended in 200 µL of phosphate-buffered saline (PBS, pH 7.2), yielding a tenfold concentration relative to the initial volume. The purified tailocins were stored at 4 °C until further analysis.

The concentration of tailocin particles was determined using a semi-quantitative spot test assay, as described earlier^9^. Briefly, Petri dishes containing TSA were overlaid with soft top agar (STA). Before pouring, the soft top agar was cooled to 48 °C and inoculated (1:60) with an overnight culture of tailocin-sensitive strain *M. paradisiaca* IFB 0117 (NCPPB 2511)^14^. The samples tested for tailocin titer were serially twofold diluted in PBS (pH 7.2). Then, in duplicates, 2 µL of each tested suspension was spotted on the plates inoculated with *M. paradisiaca*. After overnight incubation at 28 °C, the highest dilution of tailocins causing the formation of clear zones (plaques) on the bacterial lawn was determined. Two-fold tailocin dilutions were prepared and tested to enhance resolution. Since the tested method for determining tailocin concentration is based on assessing their activity, the results were expressed as relative activity in arbitrary units (AU), with 1 AU being defined as equal to the highest dilution that still caused a visible plaque on the lawn of sensitive bacterial strain (*Musicola paradisiaca* strain IFB 0117 (NCPPB 2511)). Consequently, the results in the graphs are presented as log₂AU.

### Effect of mitomycin C concentration on P2D1 yield

To determine whether the concentration of mitomycin C affects the induction and the final yield of the P2D1 tailocins in *D. dadantii* strain 3937, we tested eleven concentrations of mitomycin C. *D. dadantii* strain 3937 was grown for 16 h in 10 mL of TSB at 28 °C with shaking (120 rpm). The cultures were then diluted 1:40 in fresh TSB, incubated under the same conditions for 2.5 h (to approx. 7.5 CFU mL^− 1^, turbidity 0.8-1.0 McFarland), and then spiked with 0.1, 0.2, 0.3, 0.4, 0.5, 1.0, 1.5, 2.0, 3.0, 4.0, or 10 µg mL^− 1^ of mitomycin C (Table S1). Following the addition of the inducer, the resulting bacterial cultures were further incubated at 28 °C for 6 h with shaking (120 rpm). In each case, tailocin titer was estimated as described above. The experiment was independently conducted four times with two replicates each, and the results were averaged.

### Effect of mitomycin C on the viability of *D. dadantii* 3937 cells

*D. dadantii* strain 3937 was cultured in TSB medium for 16 h at 28 °C with shaking (120 rpm). The overnight cultures were rejuvenated (1:40) in 250 mL of fresh TSB medium, and the incubation was continued under the same conditions for 2.5 h (to approx. 7.5 CFU mL^− 1^, turbidity 0.8-1.0 McFarland). Subsequently, mitomycin C was added to a final concentration of 1 µg mL^− 1^, and the cultures were continued under the same conditions. Samples were collected at various time points during the incubation: 2.5 h before mitomycin C addition, at the time of inducer administration (T = 0), and at 0.5, 1, 2, 4, 6, 8, and 24 h post-treatment. The control sample consisted of bacterial cultures not subjected to mitomycin C treatment. To quantify the number of cells, all samples were serially diluted tenfold in PBS (Sigma-Aldrich, pH 7.2), and 10 µl aliquots of the dilutions were plated on TSA plates by spotting as described earlier^23^. The plates were dried under a laminar flow until the liquid was fully absorbed and incubated at 28 °C for 20 h. Following incubation, the number of colonies was counted, and the average colony-forming units (CFU) per milliliter of the original culture were calculated. The experiment used two biological replicates, with each containing three technical replicates.

### Yield of P2D1 Tailocins at different time points after induction

To examine the changes in P2D1 concentration following the addition of the inducing agent, strain *D. dadantii* 3937 was grown for 16 h in 10 mL of TSB at 28 °C with shaking (120 rpm). The cultures were then diluted 1:40 in fresh TSB and incubated under the same conditions for an additional 2.5 h. To induce the production of tailocins, bacterial cultures were spiked with 1 µg mL^− 1^ of mitomycin C, and the resulting culture was incubated under the same conditions for another 24 h. Culture samples were collected at T = 0 (addition of mitomycin C to *D. dadantii* strain 3937 culture) and after 0.5, 1, 2, 4, 6, 8, and 24 h after induction. In each case, tailocin titer was estimated as described above using the semi-quantitative spot test with *M. paradisiaca* IFB 0117 (NCPPB 2511) as a reporter strain. The experiment was repeated three times, and the results were averaged.

### Expression of genes involved in P2D1 synthesis with RT-qPCR

Expression of the fiber, tube, sheath, and reference genes *lpxC* and *rplU* (Fig. S1) was analyzed by RT-qPCR at 0, 1, 2, and 4 h after mitomycin C induction, alongside untreated samples collected at the same time points. Three independent biological replicates were analyzed for each condition and time point.

#### Isolation of RNA

Total RNA from bacterial cultures was isolated using the RNeasy Protect Bacteria Mini Kit (QIAGEN) according to the manufacturer’s protocol involving proteinase K treatment. The processed samples included the cultures of *D. dadantii* 3937 grown as described for determining the activity of tailocin inducers, harvested immediately before the addition of mitomycin C (T = 0), and at 1, 2, and 4 h after treatment. Samples untreated with mitomycin C were processed in the same way as a control. For time points 0, 1, and 2 h post-induction, 5 ml of the culture was mixed with RNAprotect Bacteria Reagent (QIAGEN) at a 1:2 ratio, and the cells were pelleted (10 min., 5000 RCF, 22 °C) for isolation of RNA. For mitomycin-treated samples collected after 4 h, due to the loss in viable cell count, a larger volume (50 ml) of the culture was harvested, pelleted (3 min, 8000 RCF, 22 °C), resuspended in 500 µl of PBS buffer, and immediately mixed with 1 ml of the RNAprotect Bacteria Reagent. The increased culture volume processed for timepoint T = 4 h did not apply to the control (untreated) samples. The purified RNA was treated with the TURBO DNA-free Kit (Thermo Fisher Scientific) to remove potential genomic DNA contamination. The integrity of RNA was verified by agarose gel electrophoresis, and its concentration was determined using NanoDrop 2000 (Thermo Fisher Scientific). The samples were stored at -80 °C for further use.

#### cDNA synthesis, real-time qPCR, and expression analysis

RNA was reverse transcribed into cDNA using the Transcriptor First Strand cDNA Synthesis Kit (Roche) with random hexamer primers and with the optional denaturation step. For each reaction, 500 ng of RNA was used as a template. Real-time qPCR was performed on the CFX96 instrument (Bio-Rad) using Power SYBR Green PCR Master Mix (Thermo Fisher Scientific), as described before^24^. The template cDNA was diluted 1:4. All primers used in the experiment were designed using Primer3Plus^25^ (Fig. S2, Fig. S3, and Table S2). Primer specificity was verified by agarose gel electrophoresis and melt curve analysis (Fig. S1, Fig S2). Primer efficiency was established based on serial dilutions of post-PCR products as a template (Fig. S3). Each biological replicate was analyzed in two technical replicates. A sample maximization design was employed, in which all samples for a given gene were run on the same qPCR plate to minimize inter-run variability. Relative gene expression was quantified using the ΔΔCq method^26^implemented in the gene expression analysis module of CFX Maestro 2.3 software (Bio-Rad). Target gene expression was normalized to the reference genes *lpxC* and *rplU*, previously validated as stable in *D. dadantii*^27^. Expression levels for each target gene were compared to the 0-hour control sample (collected immediately prior to inducer addition). Samples collected at 1, 2, and 4 h included both mitomycin C-treated and untreated conditions, enabling evaluation of time-dependent changes in tailocin structural gene expression and comparison between treated and untreated cells. Gene expression results relative to the control group (prior to induction) were presented both as log₂ fold changes and as values converted to linear scale, to facilitate interpretation.

### Effect of antibiotics on P2D1 Tailocin yield

To test whether chemicals other than mitomycin C can induce the production of P2D1 tailocins in *D. dadantii* strain 3937, four antibiotics with different modes of action were selected and used in a model experiment: chloramphenicol – inhibiting protein synthesis, ampicillin – that inhibits bacterial cell wall synthesis, ciprofloxacin, and norfloxacin – both inhibiting DNA replication and repair. *D. dadantii* strain 3937 was grown for 16 h in 10 mL of TSB at 28 °C with shaking (120 rpm). The cultures were then diluted 1:40 in fresh TSB and incubated under the same conditions for an additional 2.5 h. To induce the production of tailocins, bacterial cultures were spiked with either chloramphenicol (final concentration: 4 µg mL^− 1^), ampicillin (final concentration: 0.004 µg mL^− 1^), ciprofloxacin (final concentration: 0.016 µg mL^− 1^) and norfloxacin (final concentration: 0.016 µg mL^− 1^)(Table S1). As a positive control and known P2D1 inducer, mitomycin C (final concentration: 1 µg mL^− 1^) was used. After adding putative inducers, the resulting bacterial cultures were further incubated at 28 °C for 6 h with shaking (120 rpm). In each case, tailocin titer was estimated as described above using the semi-quantitative spot test with *M. paradisiaca* IFB 0117 (NCPPB 2511) as a reporter strain. The obtained results were expressed as relative activity in arbitrary units (AU) as described above. The experiment was independently conducted four times, containing two technical replicates each, and the results were averaged.

### Effect of hydrogen peroxide on P2D1 Tailocin yield

To assess the effect of hydrogen peroxide on tailocin induction in *D. dadantii* 3937 and to compare it with the bacterial cell response induced by mitomycin C, an experiment was conducted following similar conditions as those described above to test the effect of varying concentrations of mitomycin C. The only difference in treatment was that four concentrations of hydrogen peroxide (0.5, 1, 5, and 10 mM) were tested instead of mitomycin C, at 6 h post induction (Table S1). A single concentration of mitomycin C (1 µg mL^− 1^) and an untreated bacterial culture were positive and negative controls, respectively. The yield of P2D1 particles obtained following each treatment was expressed in relative units (AU) as described above. The experiment was conducted three times, each time with two technical replicates.

### Effect of the type of growth medium on P2D1 Tailocin yield

To test whether the type and composition of the growth medium affect the production of P2D1 tailocins, three different media were tested: M9 minimal medium with 0.4% glucose, PDB (rich medium with potato extract and glucose), and TSB (rich medium with peptone and glucose). *D. dadantii* strain 3937 was grown for 16 h in 10 mL of TSB at 28 °C with shaking (120 rpm). The cultures were diluted 1:40 in fresh TSB, PDB, or M9 + 0.4% glucose medium and incubated under the same conditions for 2.5 h. To induce the production of tailocins, bacterial cultures were spiked with 1.0 µg mL^− 1^ of mitomycin C. Following the addition of the inducer, the resulting bacterial cultures were further incubated at 28 °C for 6 h with shaking (120 rpm). In each case, tailocin titer was estimated as described above using the semi-quantitative spot test with *M. paradisiaca* IFB 0117 (NCPPB 2511) as a reporter strain. The experiment was independently repeated three times, with two replicates each, and the results were averaged.

## Results

### P2D1 Tailocin production is dependent on the concentration of mitomycin C inducer

**Fig. 1:**
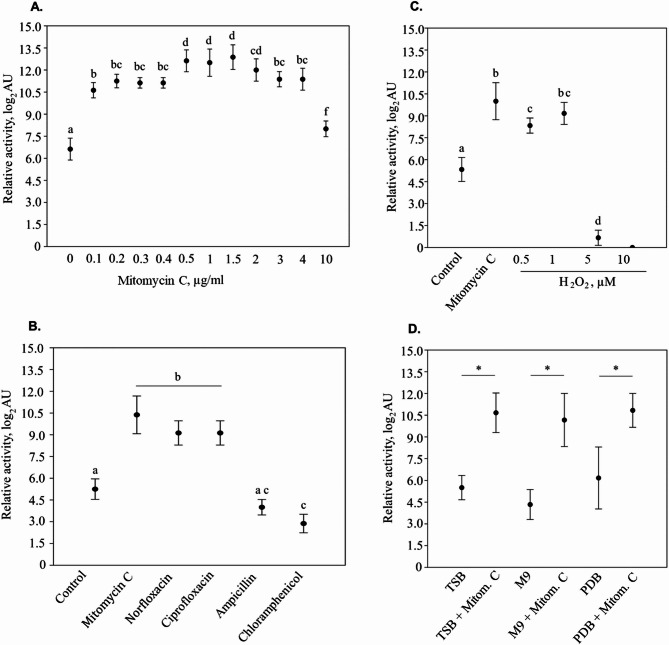
Production of tailocins by *D. dadantii* 3937 depending on the applied concentration of mitomycin C, the growth medium, and the type of inducer. Tailocin titer was expressed as relative tailocin activity in arbitrary units (AU), with 1 AU defined as the reciprocal of the highest dilution that caused a visible plaque on a lawn of susceptible strain *M. paradisiaca* IFB 0117 (NCPPB 2511). In all panels, data points show mean values, and error bars represent standard deviations. Statistically significant differences between groups for analyses in panels A and C were determined using Tukey’s pairwise comparison^43^followed by Copenhaver Holland post hoc analysis^44^while for analyses in panels B and D, these were determined by Kruskal-Wallis test^45^followed by Dunn’s post hoc test^46^. In all panels, groups labeled with the same letter are not significantly different (α = 0.05). Panel (**A**) depicts the titer of P2D1 tailocins depending on the applied concentration of mitomycin C. Panel (**B**) shows tailocin titer when testing the inducive potential of selected antibiotics: norfloxacin 0.016 µg mL^− 1^; ciprofloxacin 0.016 µg mL^− 1^; ampicillin 0.004 µg mL^− 1^; chloramphenicol 4 µg mL^− 1^. Treatment with mitomycin C (1 µg mL^− 1^) was used as a reference. Control – culture with no potential inducer added. Panel (**C**) shows tailocin yield following induction with different concentrations of hydrogen peroxide: 0.5, 1, 5, and 10 mM, with mitomycin treatment as reference (1 µg mL^− 1^). **Control** – culture with no potential inducer added. Panel (**D**) shows tailocin yield in different growth media when induced with mitomycin C (1 µg mL^− 1^): TSB – Trypticase Soya Broth; M9 – M9 minimal medium with 0.4% glucose; PDB – Potato Dextrose Broth.

*D. dadantii* 3937 was reported to produce P2D1 tailocins at a baseline level of only about ca. 6.5 log_2_AU when grown in TSB in the absence of an inducer^14^. However, production was greatly stimulated following treatment with 0.1 µg mL^− 1^ of mitomycin C^14^. Here, we established the effectiveness of mitomycin C induction over a range of concentrations (0.1–10 µg mL^− 1^). Although mitomycin C significantly upregulated P2D1 tailocin production at all concentrations tested, the highest yield was observed at concentrations between 0.5 and 1.5 µg mL^− 1^. At these concentrations, activity levels approached 12.5 log_2_AU, an approximately 64-fold increase relative to that in untreated cells (Fig. 1A).

### **Mitomycin C causes a decline in viable*D. dadantii*3937 cells that is** caused by the accumulation of P2D1 Tailocins

**Fig. 2:**
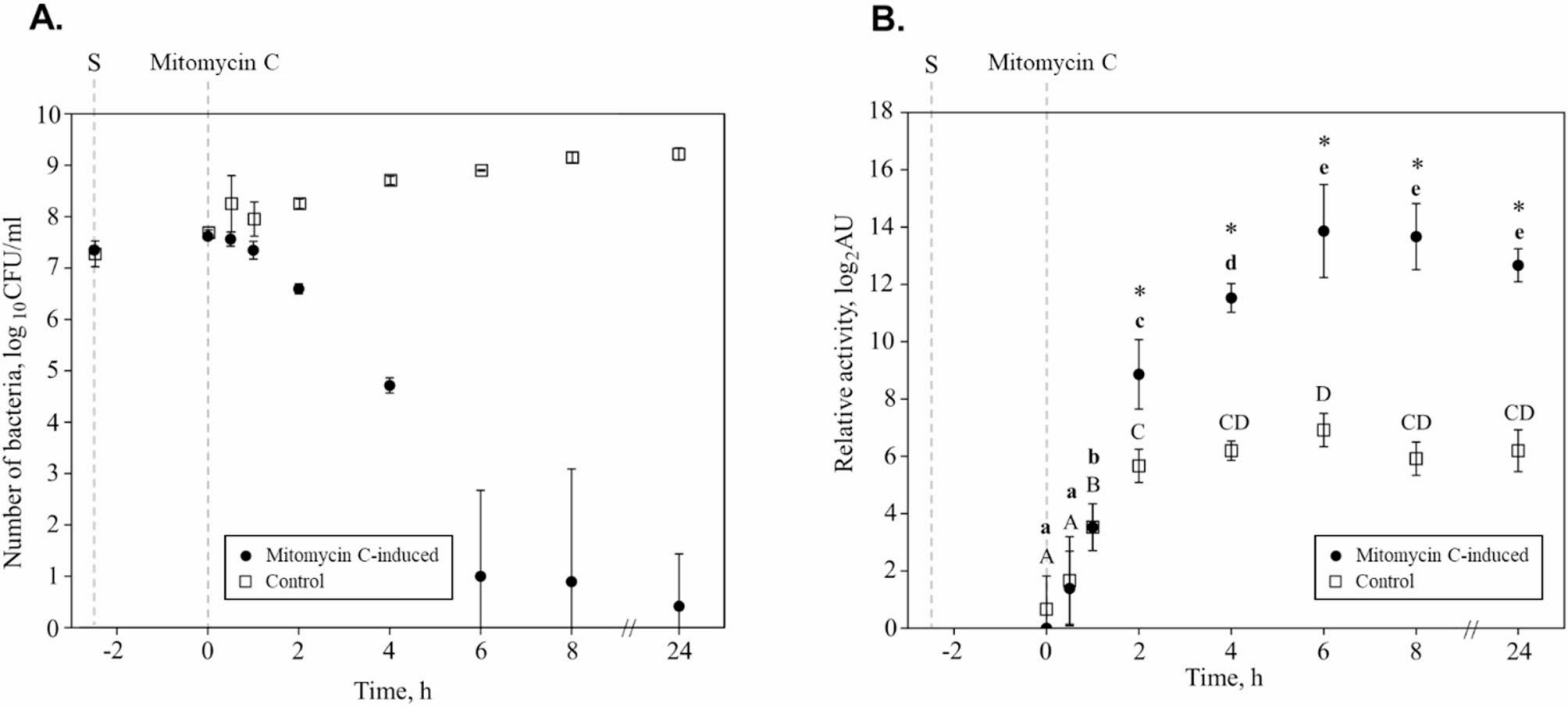
Tailocin yield and viable bacterial cell count as a function of time following the addition of inducer. (**A**) Survival of *D. dadantii* strain 3937 over time, measured in colony-forming units (CFU). Data points represent the viable bacterial cell count in cultures treated with mitomycin C at a concentration of 1 µg mL^− 1^. The counts are shown at various intervals, illustrating the decline in bacterial viability as the cells produce and release tailocins.(**B**) The titer of P2D1 tailocins isolated from the culture of *D. dadantii* strain 3937 at different time points (0–24 h) post induction with mitomycin C (1 µg mL^− 1^). The x-axis indicates the incubation time of bacterial cells with the inducer, after which tailocins were harvested for activity against *M. paradisiaca* IFB 0117 (NCPPB 2511). Cultures unexposed to mitomycin C were used as controls. Points represent the mean activity (mean AU) of measurements from 3 independent experiments. Error bars indicate standard deviation ranges. S represents the start of the culture, and Mitomycin C indicates the time of spiking the culture with mitomycin C. In panel (B), significant differences between Mitomycin C-treated samples collected at different time points were determined using the Mann-Whitney U test^47^. Groups labeled with the same letter are not significantly different (α = 0.05). Small letters are used for Mitomycin C-treated samples and capital letters for control. Asterisks (*) indicate statistically significant differences (*p* < 0.05; Mann-Whitney U test^47^ between experimental and control samples collected at the same time points.

The impact of mitomycin C (1 µg mL^− 1^) on the growth and survival of *D. dadantii* strain 3937 in TSB medium was evaluated. In the control culture, which was not exposed to mitomycin C, the number of viable bacterial cells increased over 34-fold during a 24-hour incubation period (rising from 7.7 to 9.2 log CFU mL^− 1^) (Fig. 2A). However, in the presence of mitomycin C, bacterial survival dropped sharply. Within 4 h after the addition of mitomycin, the number of viable cells decreased approximately 800-fold (from 7.6 to 4.7 log CFU mL^− 1^). This decline was even more pronounced by 6 h after mitomycin addition, with viable cell numbers dropping 3.6 million-fold to just 10 CFU mL^− 1^. By the end of the 24-hour incubation with mitomycin C, the bacterial population was nearly eradicated, with only about 4 CFU mL^− 1^.

### P2D1 Tailocin production reaches a maximum level six hours after induction with mitomycin C

Significant increases in P2D1 production in mitomycin C-treated samples over that in the untreated control were seen within 2 h after mitomycin treatment: tailocin abundance increased 9-fold (to 8.86 log₂AU) relative to the control (5.67 log₂AU) (Fig. 2B). The highest P2D1 titer was observed 6 h after mitomycin C addition, reaching 13.9 log₂AU, representing about a 123-fold increase compared to the untreated control. No further increases in P2D1 tailocin yield upon mitomycin C -treatment were observed in samples collected from 6 to 24 h after induction. This indicates that after reaching its maximum titer, the number of P2D1 tailocin particles remained stable (Fig. 2B).

### Expression of genes encoding P2D1 structural genes peaks two hours after induction

**Fig. 3:**
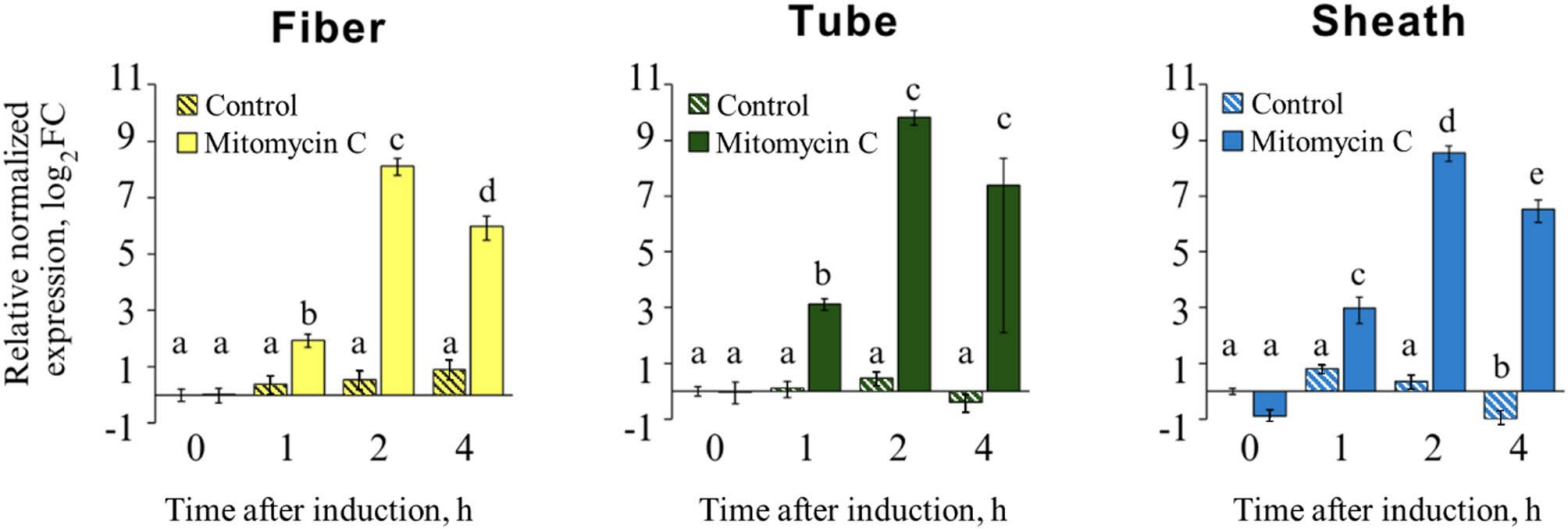
Expression of P2D1-encoding structural genes in *D. dadantii* 3937 at different time points following induction with mitomycin C. The graphs depict the relative expression levels of three target genes: fiber (DDA3937_RS12070), tube (DDA3937_RS12115), and sheath (DDA3937_RS12110), in samples collected after specified incubation times following the addition of mitomycin C (1 µg mL^[- [1^). Control samples consist of bacteria that were not treated with the antibiotic. The fold change (log2) in gene expression for the target genes was calculated relative to the control samples collected at time 0. Statistically significant differences among the experimental groups were determined using one-way ANOVA^48^, followed by Tukey’s Honest Significant Difference post hoc analysis^49^. Groups marked with the same letter do not differ significantly (α = 0.05).

The expression of three genes encoding structural proteins of P2D1 tailocin (responsible for forming the sheath, tube, and tail fiber) was analyzed using RT-qPCR to monitor the temporal pattern of tailocin gene expression after induction. Gene expression was compared between *D. dadantii* cells treated with mitomycin C (1 µg mL^− 1^) and untreated control cells. In the control cells to which no inducer was added, no significant changes in gene expression were observed over time, except for a slight increase in the expression of the sheath protein gene at 4 h (Fig. 3). In contrast, cells exposed to mitomycin C showed a marked increase in the expression of all tailocin genes. After 1 h, the expression of the genes encoding the sheath, tube, and fiber proteins increased by approximately 15-, 9-, and 4-fold, respectively. Peak expression was seen 2 h after induction, with the tube protein gene exhibiting a 655-fold increase, while the sheath and fiber genes increased by 295- and 191-fold, respectively, compared to the control (Fig. 3, Table S3). By 4 h, the expression of all three tailocin genes had numerically declined, with the decrease being statistically significant for the fiber and sheath genes (Fig. 3).

### P2D1 Tailocins are induced by antibiotics that affect the replication and repair of bacterial genomic DNA

We examined the impact of selected antibiotics (viz. ampicillin, ciprofloxacin, norfloxacin, and chloramphenicol) on the production of P2D1 tailocins. Notably, norfloxacin and ciprofloxacin significantly increased tailocin production, with tailocin activity levels rising 14-fold compared to the control (untreated culture). These results were statistically comparable to the induction of P2D1 tailocins conferred by mitomycin C. In contrast, chloramphenicol caused a five-fold decrease in P2D1 production, whereas ampicillin addition did not affect P2D1 tailocin production (Fig. 1B).

### P2D1 Tailocins are inducted by hydrogen peroxide

Supplementation of *D. dadantii* 3937 culture with hydrogen peroxide significantly affected P2D1 tailocin production, as evidenced by changes in bactericidal activity against *M. paradisiaca* IFB 0117 (NCPPB 2511) (Fig. 1C). The highest mean induction was observed after adding H₂O₂ to the culture to a concentration of 1 mM, increasing bactericidal activity to an average of 9.2 log₂AU, making it 14.3 times higher than the control (5.3 log₂AU). Adding H₂O₂ to a concentration of 1 mM conferred a similarly high level of tailcin production as 1 µg mL^− 1^ of mitomycin C. H₂O₂ at 0.5 mM increased activity 8-fold (to 8.3 log₂AU). In contrast, tailocin production in cultures treated with the highest concentrations of hydrogen peroxide (5 and 10 mM H₂O₂) was negligible (Fig. 1C). Cell viability measurements revealed that treatment with 1 mM hydrogen peroxide caused only a modest reduction in viable cell counts in comparison to the control (approx. 3.5-fold) 15 min after exposure, followed by an increase in viability at the 6- and 24-hour time points. In contrast, exposure to 10 mM hydrogen peroxide led to a tenfold decrease in viability within 15 min (from approximately 7.7 to 6.6 log CFU mL-1), with cell counts remaining 4 log units lower than those observed for 1 mM treatment at both 6 and 24 h post-treatment (poor recovery). Treatment with 5 mM H₂O₂ had an intermediate effect, causing a sharp initial decline in viable cell counts – comparable to that observed with 10 mM – followed by a partial recovery, albeit slower than that seen in the 1 mM condition.

### The type of the growth medium affects neither the basal level nor the induction of P2D1 Tailocins

We found that the type of microbiological medium used during the cultivation of the *D. dadantii* 3937 strain did not affect the production level of P2D1 tailocins (mean 5.3 log_2_AU). This was true both for the constitutive tailocin production in the absence of any inducers as well as upon the addition of mitomycin C (mean 10.6 log_2_AU) (Fig. 1D), as no statistically significant differences (α = 0.05) were observed within the cells in the various unmodified media or between multiple media to which mitomycin C had been added.

## Discussion

**Fig. 4:**
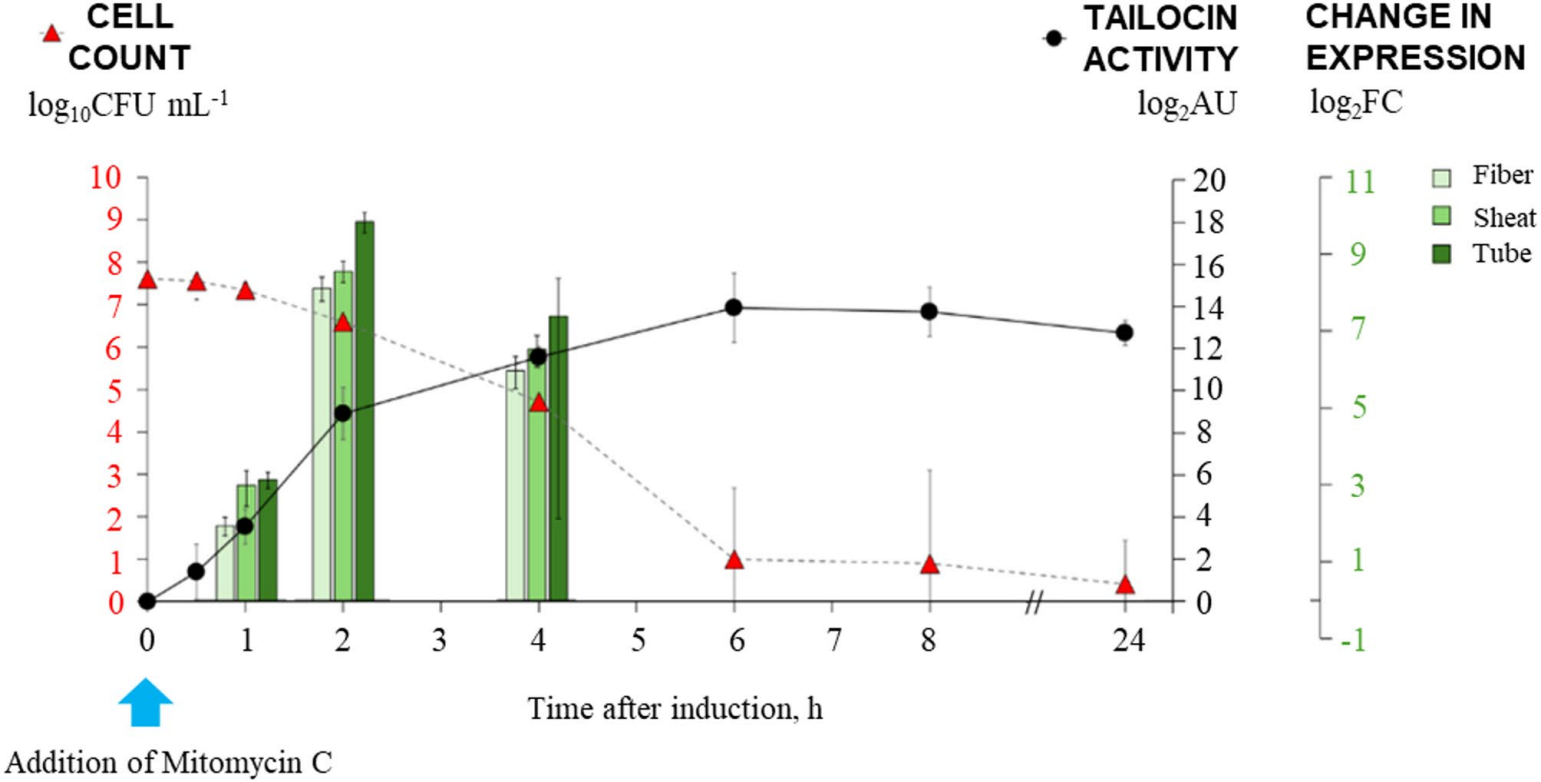
Temporal response of *D. dadantii* 3937 to mitomycin C. The figure illustrates the dynamics of strain 3937 in response to stress induced by mitomycin C. The graph presents the number of viable bacterial cells producing P2D1 tailocins, the relative activity of the produced tailocins against *M. paradisiaca* IFB 0117 (NCPPB 2511), and the relative expression levels of genes encoding structural proteins of the tailocins. The cultures were treated with mitomycin C at a concentration of 1 µg mL^[- [1^, with the inducer added to the bacterial culture at time 0.

Although multiple studies have investigated the genetic factors associated with the production of phage tail-like particles (tailocins), wide-ranging analyses examining the interplay between tailocin inducers, induction timing, structural gene expression, and the viability of the producing bacterial cells remain scarce. Likewise, most studies have addressed the induction of tailocins only with mitomycin C and have not assessed how promiscuous tailocin production might be in the presence of other compounds that assault bacteria in natural habitats. This study has thus addressed the dynamics of the production of P2D1 tailocin and the various toxicants that would induce its production in plant-pathogenic *Dickeya* spp^28^. using the model strain *D. dadantii* 3937 ^20^ (see Fig. 4).

The marked upregulation of P2D1 production was observed across a range of inducer concentrations, indicating that the regulation of tailocin production is a quantitative rather than qualitative trait. This observation confirmed the importance of DNA damage as an inducer of this potent phage tail-like particle in *D. dadantii* 3937, as we have demonstrated earlier^14^. It also extends these findings by characterizing the dose-dependent effects of mitomycin C. Our observations align with findings on other bacteriocins, where moderate stress levels maximize bacteriocin synthesis^29^. However, the highest concentrations of mitomycin C used in our study (above 1.5 µg mL^− 1^) resulted in significantly decreased P2D1 yields. This is consistent with other studies of the impact of mitomycin on cells, which show that excessive DNA damage from mitomycin C treatment overwhelms cellular repair machinery, resulting in impaired cellular functions and even death^30^thereby reducing tailocin yield.

The induction of P2D1 tailocin with mitomycin C caused a remarkable decline in viable *D. dadantii* cells (ca. 3.5-million-fold reduction observed by 6 h after induction). Such an observation aligns well with the known canonical mechanism of tailocin production, where the synthesis and release of these particles disrupt the cell membranes and/or negatively affect other cellular processes, resulting in the rapid death of the producers^4,31^.

The dynamics of tailocin production is, therefore, correlated with the overall number of viable cells within the population capable of producing tailocins. In *Pseudomonas aeruginosa*, it has been shown that under natural conditions, less than 1% of the population spontaneously produces tailocins and undergoes lysis, whereas under artificial induction with mitomycin C, tailocin production and cell lysis become widespread and frequent^31^a phenomenon that we also observed in our study. However, it is also notable that a small fraction of induced cells in our study survived. This might be attributed to a limit on the number of cells within the population that are capable of producing tailocins. Such a limit is likely driven by a heterogeneous response to stress factors within a cell population^31^. This heterogeneous response suggests that not all cells activate tailocin production, resulting in a subset of cells that do not undergo lysis and thus survive. We speculate that our observations mirror the situation observed in colicin production, where a self-limiting regulation loop ensures efficient resource allocation during stress^32^.

Furthermore, the constant levels of tailocin abundance six hours or more after induction suggests that their production may cease once a certain threshold concentration is reached. The transcriptional activity of structural tailocin genes peaked 2 h post-induction. Then, a marked decline in mRNA levels appeared by 4 h, indicating that transcription is temporally limited. However, the concentration of tailocin particles continued to rise until 6 h, indicating that translation and particle assembly persisted transiently after the peak in transcription and that tailocin particles accumulated progressively during this period. After 6 h, particle levels plateaued. The constant levels of tailocin activity from 6 to 24 h imply that P2D1 particles are highly stable once assembled. It can be predicted that their stability in the natural environment would depend on factors such as high temperature, freeze-thaw cycles, highly acidic pH, and the presence of proteases, all of which we have shown to decrease the activity of P2D1 ^14^.

Simultaneously with the peak in tailocin production, we observed a sharp decline in cell viability between 4 and 6 h after mitomycin C treatment, with over 99.9% of the population dead at the 6-hour time point compared to T = 0. Analysis of the temporal expression of P2D1 structural genes revealed that peak transcription also occurred two hours after induction - with a significant upregulation of all P2D1 tailocin genes encoding tube, fiber, and sheath. This fast transcriptional response is characteristic of bacteriocin systems regulated by the SOS response^33^where such genes are among those induced in response to DNA-damaging agents^32,34^. Similar temporal patterns as seen here have been reported in other bacterial species, where gene expression peaks early in the SOS stress response, followed by a decline as the cells transit into a survival state^6,35^. The declining expression of tailocin genes observed 4 h or more after exposure to DNA-damaging agents may suggest that their transcriptional activation is tightly regulated. Such a synchronized response ensures that the synthesis of tailocins, such as the P2D1, does not compromise cellular integrity during prolonged stress^36^. In other microorganisms, the regulation of tailocin synthesis involves complex mechanisms that balance positive and negative regulatory factors. For instance, in *Pseudomonas aeruginosa*, tailocin production is controlled by the positive regulator PrtN and the negative regulator PrtR, with additional oversight provided by the LexA protein, which acts as a gatekeeper for the SOS response^37^. In *Stenotrophomonas maltophilia*, MpsA and MpsH function as positive regulators, while MpsR acts as a repressor under normal conditions, preventing unnecessary tailocin production^38^. The proteins and mechanisms involved in regulating the production of P2D1 tailocin in *Dickeya dadantii* and any buffering system that allows a portion of the population to survive despite the high cost of tailocin production under inducing conditions remain to be elucidated.

The induction of P2D1 by compounds other than mitomycin C (i.e., antibiotics and hydrogen peroxide) provides valuable insights into the versatility of tailocin regulation in *D. dadantii*. Perhaps not surprisingly, antibiotics such as ciprofloxacin and norfloxacin, which inhibit bacterial replication, apparently mimic the effect of mitomycin C by inducing the SOS response, as seen in other studies^39,40^. Similarly, oxidative stress caused by hydrogen peroxide conferred tailocin production in *D. dadantii* to levels similar to that mediated by mitomycin C. This latter observation supports the hypothesis that tailocin synthesis is under the control of the generalized stress response^4,13,31^. However, the significantly decreased production of P2D1 tailocins at higher hydrogen peroxide concentrations highlights the importance of stress insensitivity in modulating tailocin output^10^. Here, 1 mM hydrogen peroxide induced the highest tailocin production, likely generating sufficient stress to trigger synthesis without severely compromising cell viability. In contrast, the sharp reduction observed at 10 mM likely reflects rapid and extensive cell death. The reduced tailocin yield at 5 mM is more difficult to interpret but may result from a combination of decreased viability and – based on studies in *Escherichia coli* – suppressed metabolic activity, potentially associated with a transition into a persister-like state^41^. Lastly, as expected, chloramphenicol inhibited P2D1 production, likely by impairing protein synthesis and interference with cell wall synthesis, respectively^42^.

The lack of differences in P2D1 tailocin accumulation in several different growth media highlights the robustness and apparent environmental insensitivity of tailocin synthesis in *D. dadantii* strain 3937. These findings contrast with studies on other phage tail-like particles, where medium composition strongly influenced tailocin yield due to the differences in nutrient availability or pH^6,9^. The medium independence of P2D1 production suggests that tailocin regulation is predominantly governed by the activation of cellular stress pathways rather than other factors.

Our study gives new insights into the events during the assembly and release of P2D1 tailocin in *D. dadantii* and probably to tailocin synthesis in other closely related plant pathogenic bacteria. Other studies have yet to provide detailed insights into the sequential events of tailocin assembly and release under various inducing conditions. The tight linkage between stress and response and tailocin production seen in this study supports the need for further studies of the regulation of tailocin production to better link it to inter- and intraspecies competition in natural habitats where such stresses are common.

## Supplementary Information

Below is the link to the electronic supplementary material.

Supplementary Material 1

## Data Availability

Data generated or analyzed during this study are included in this article (including its Supplementary Information files). These data can also be obtained from the corresponding author and shared freely upon reasonable request. Correspondence and requests for materials should be addressed to Robert Czajkowski (robert.czajkowski@ug.edu.pl) and/or to Dorota M. Krzyzanowska (dorota.krzyzanowska@ug.edu.pl).
