# Supplementary material for: Stress-driven temporal production of phage tail-like particles (tailocins) in Dickeya dadantii strain 3937
Source: Sci Rep. 2025 Jul 26;15:27234. doi: 10.1038/s41598-025-13158-1 (PMC12297226; doi:10.1038/s41598-025-13158-1)
Supplement: Supplementary file 1 — Supplementary Material 1 [file 41598_2025_13158_MOESM1_ESM.docx]

**SUPPLEMENTARY INFORMATION**

*to research article*

**Stress-driven temporal production of phage tail-like particles (tailocins) in *Dickeya dadantii* strain 3937**

Marta Sobolewska ^a^, Dorota M. Krzyżanowska ^b,^ *, Marcin Borowicz ^a^, Robert Czajkowski ^a,^ *

^a^ Laboratory of Biologically Active Compounds, Intercollegiate Faculty of Biotechnology of the University of Gdańsk and Medical University of Gdańsk, University of Gdańsk, A. Abrahama 58, 80-307 Gdańsk, Poland;

^b^ Laboratory of Plant Microbiology, Intercollegiate Faculty of Biotechnology of the University of Gdańsk and Medical University of Gdańsk, University of Gdańsk, A. Abrahama 58, 80-307 Gdańsk, Poland;

* Correspondence:

Robert Czajkowski (robert.czajkowski@ug.edu.pl; phone: 0048 58 523 6333)

Dorota M. Krzyżanowska (dorota.krzyzanowska@ug.edu.pl: 0048 58 523 6316)

# Supplementary Figures

## **Figure S1. Specificity of primers applied in real-time qPCR.** Agarose gel electrophoresis (2%) depicting single PCR products of the expected sizes for each of the analyzed target genes. L – GeneRuler™ 100bp DNA Ladder Plus (Thermo Fisher Scientific). nc – no template negative control; DNA staining was done using Novel Juice (GeneDireX). The image was captured with a ChemiDoc XSR (Bio-Rad) and processed using Image Lab software.


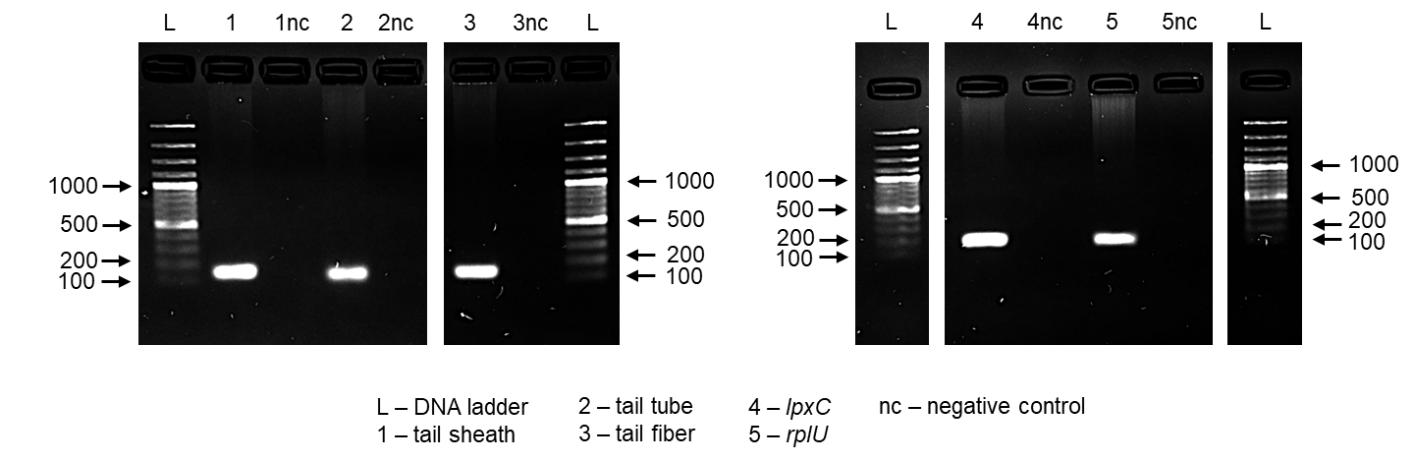


## **Figure S2. Melt curve analysis.** Melt curve analysis (55-95 ºC with 0.5 ºC increment every 5 seconds) for the amplicons of each target gene, demonstrating that each pair of PCR primers produces a single peak. The gene loci corresponding to the designated targets are provided in Table S2.


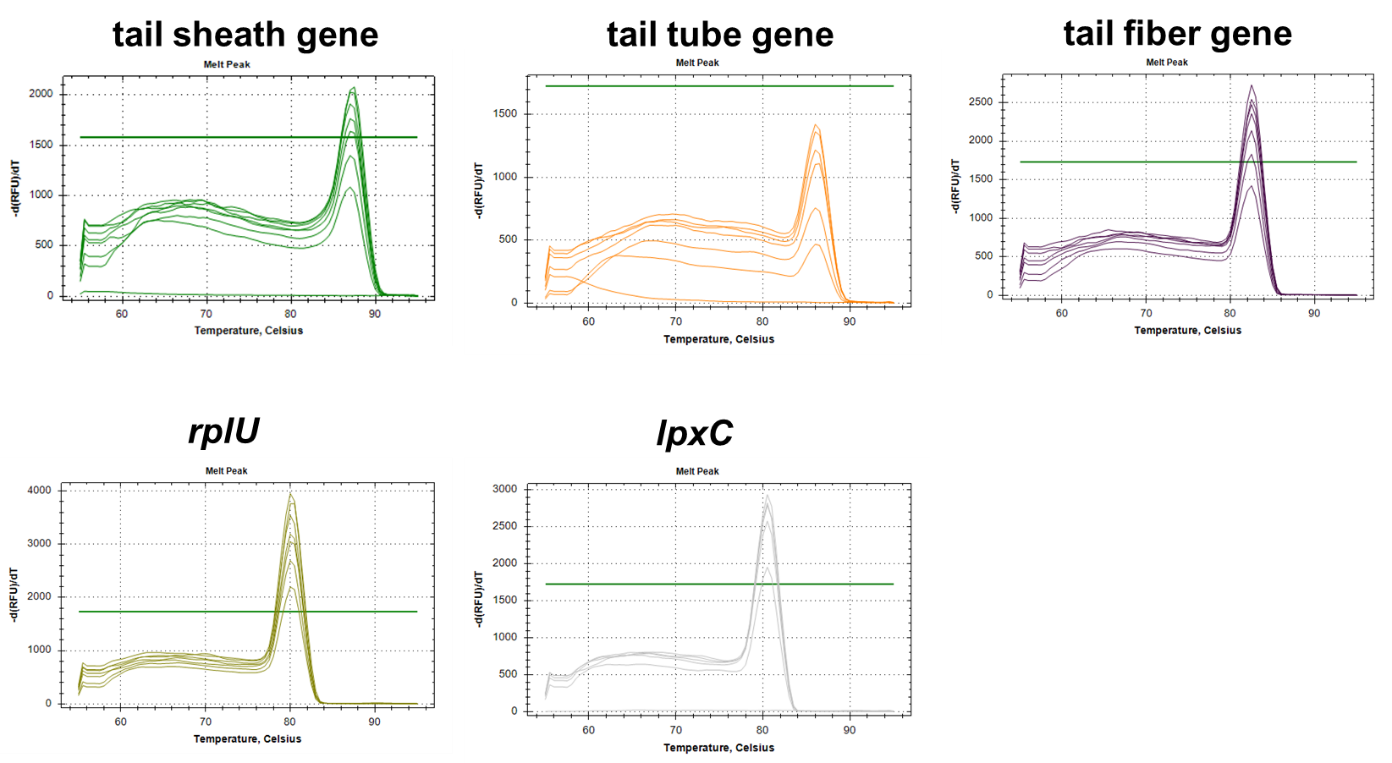


## **Figure S3. Standard curves for the estimation of PCR efficiency.** Standard curves were used to determine PCR efficiency for the reference genes (*rplU* and *lpxC*) and the target genes (tail sheath gene, tail tube gene, and tail fiber gene). The gene loci corresponding to the designated targets are provided in Table S2.


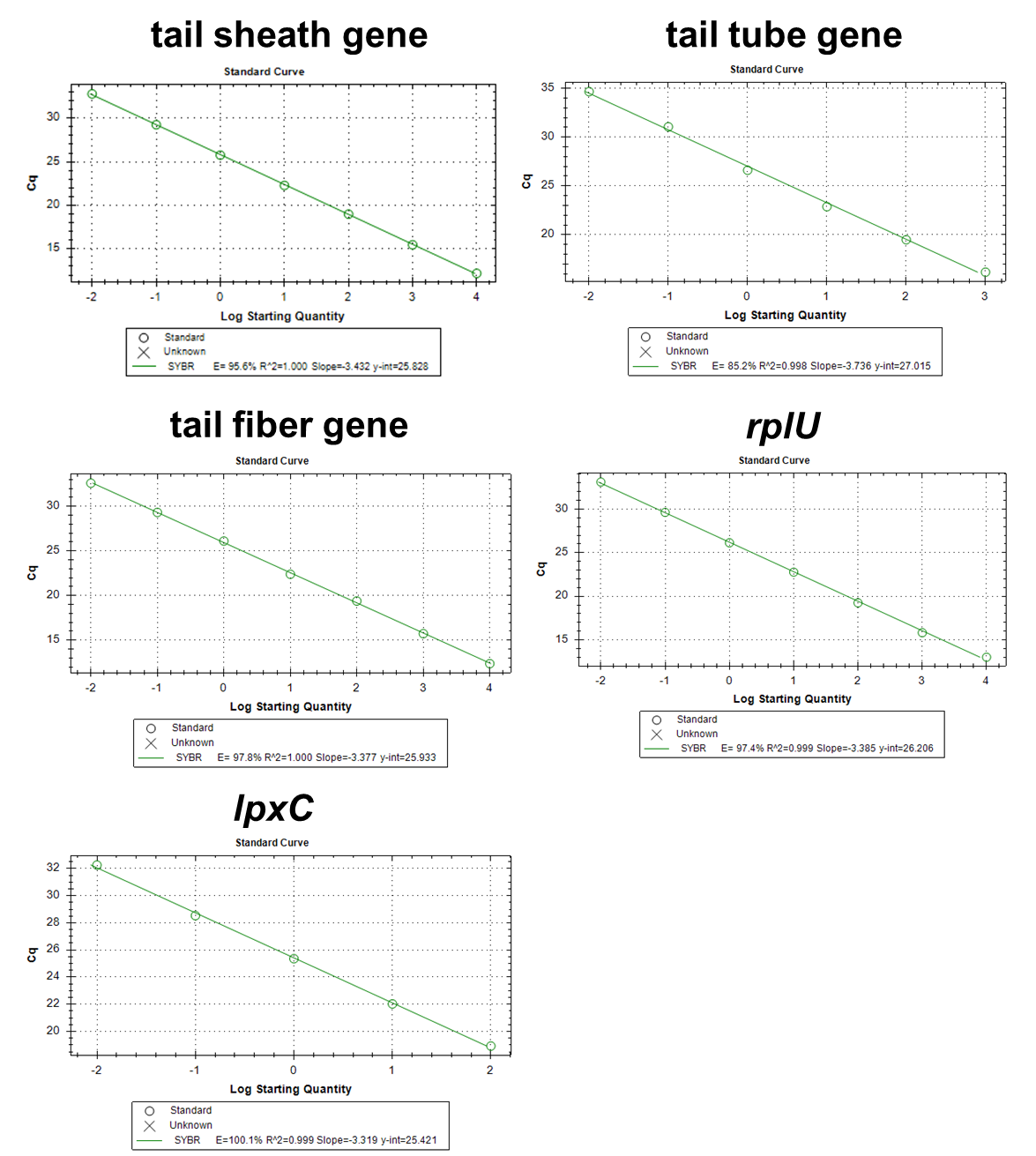


# Supplementary Tables

## Table S1. Conditions for the induction of tailocin production.

| **Experiment** | **Inducer** | **Concentration** | **Unit** | **Incubation time, h** |
| --- | --- | --- | --- | --- |
| Effect of mitomycin C concentration on tailocin yield | Mitomycin C | 0.1; 0.2; 0.3; 0.4; 0.5; 1; 1.5; 2; 3; 4; 10 | µg mL^-1^ | 24 |
| Tailocin yield at different time points after induction | Mitomycin C | 1 | µg mL^-1^ | 0; 0.5; 1; 2; 4; 6; 8; 24 |
| Potential of selected inducers to induce tailocins | Mitomycin C | 1 | µg mL^-1^ | 6 |
|  | Chloramphenicol | 4 | µg mL^-1^ | 6 |
|  | Ampicillin | 0.004 | µg mL^-1^ | 6 |
|  | Ciprofloxacin | 0.016 | µg mL^-1^ | 6 |
|  | Norfloxacin | 0.016 | µg mL^-1^ | 6 |
|  | Hydrogen peroxide | 0.5; 1; 5; 10 | mM | 6 |

## Table S2. Primers designed and used in this study.

| **Product of the target locus**  **(gene name)** | **GenBank locus tag**  **(in genome**  **NC_014500.1)** | **Primer name** | **Primer sequence** | **Amplicon length (bp)** | **Primer efficiency**  **(%)** |
| --- | --- | --- | --- | --- | --- |
| Sheath | DDA3937_RS12110 | Sht_3937_F | TGATCTGGCCGGACTTTATC | 134 | 95.6 |
|  |  | Sht_3937_R | GACGTTGGACAGGGTTTTGT |  |  |
| Tube | DDA3937_RS12115 | Tub_3937_F | GGTGATGGAATGGAACATGG | 116 | 85.2 |
|  |  | Tub_3937_R | TGTCGTCACGCTGGTAAGAG |  |  |
| Fiber | DDA3937_RS12070 | Fib_3937_F | GATCCTGATTGTCAGCACGA | 125 | 97.8 |
|  |  | Fib_3937_R | TGAGTTGAGTGTCGGCGTAG |  |  |
| UDP-3-O-acyl-N-acetylglucosamine deacetylase  (*lpxC*) | DDA3937_RS18015 | LpxC_3937_F | GCACCTGAAATTCCGATCAT | 142 | 100.1 |
|  |  | LpxC_3937_R | CCCATTTGTCACCGTCTTCT |  |  |
| 50S ribosomal protein L21  (*rplU*) | DDA3937_RS02990 | RplU_3937_F | ATGTACGCGGTTTTCCAAAG | 124 | 105.5 |
|  |  | RplU_3937_R | CAACCATCAGAACCTGGTCA |  |  |

## Table S3. Expression of structural genes encoding P2D1 at different time points following induction.

|  |  | **Control** | | | **Mitomycin C treatment** | | |
| --- | --- | --- | --- | --- | --- | --- | --- |
| **Gene**  **(locus^A^)** | **time (h)** | **Exp.** | **Exp. SD** | **log_2_ of Exp.** | **Exp.** | **Exp. SD** | **log_2_ of Exp.** |
| **Fiber** | 0 | 1.00 | 0.15 | 0.00 | 1.01 | 0.18 | 0.01 |
| RS12070 | 1 | 1.30 | 0.31 | 0.38 | 3.85 | 0.62 | 1.94 |
|  | 2 | 1.45 | 0.34 | 0.53 | 277.05 | 58.02 | 8.11 |
|  | 4 | 1.87 | 0.48 | 0.90 | 62.66 | 17.56 | 5.97 |
| **Sheath** | 0 | 1.00 | 0.07 | 0.00 | 0.54 | 0.09 | -0.89 |
| RS12110 | 1 | 1.74 | 0.18 | 0.80 | 7.90 | 2.47 | 2.98 |
|  | 2 | 1.27 | 0.22 | 0.35 | 374.81 | 70.33 | 8.55 |
|  | 4 | 0.51 | 0.11 | -0.96 | 91.84 | 24.95 | 6.52 |
| **Tube** | 0 | 1.00 | 0.12 | 0.00 | 0.99 | 0.26 | -0.01 |
| RS12115 | 1 | 1.07 | 0.21 | 0.10 | 8.78 | 1.25 | 3.13 |
|  | 2 | 1.39 | 0.23 | 0.48 | 910.87 | 162.64 | 9.83 |
|  | 4 | 0.76 | 0.16 | -0.40 | 166.36 | 162.04 | 7.38 |

Exp. – normalized relative expression; Each value is averaged from 3 biological replicates; reference genes: *lpxC, rplU*

^A^ Locus tag in the genome of *D. dadantii* 3937 (NC_014500.1); shared prefix: DDA393
